# Williams Syndrome neuroanatomical score associates with *GTF2IRD1* in large-scale magnetic resonance imaging cohorts: a proof of concept for multivariate endophenotypes

**DOI:** 10.1038/s41398-018-0166-y

**Published:** 2018-06-08

**Authors:** Chun Chieh Fan, Andrew J. Schork, Timothy T. Brown, Barbara E. Spencer, Natacha Akshoomoff, Chi-Hua Chen, Joshua M. Kuperman, Donald J. Hagler, Vidar M. Steen, Stephanie Le Hellard, Asta Kristine Håberg, Thomas Espeseth, Ole A. Andreassen, Anders M. Dale, Terry L. Jernigan, Eric Halgren, Pediatric Imaging, Neurocognition and Genetics Study, Alzheimer’s Disease Neuroimaging Initiative

**Affiliations:** 1 Department of Cognitive Science, University of California San Diego, 9500 Gilman Drive, La Jolla, CA 92093 USA; 2 Center for Multimodal Imaging and Genetics, School of Medicine, University of California San Diego, 9452 Medical Center Drive, La Jolla, CA 92093 USA; 3 Institute for Biological Psychiatry, Mental Health Center Sct. Hans, Capital Region of Denmark, Roskilde, Denmark; 4 Department of Neurosciences, School of Medicine, University of California San Diego, 9500 Gilman Drive, La Jolla, CA 92037 USA; 5 Center for Human Development, University of California San Diego, 9500 Gilman Drive, La Jolla, CA 92093 USA; 6 Department of Radiology, University of California San Diego, School of Medicine, 9500 Gilman Drive, La Jolla, CA 92037 USA; 7 NORMENT, KG Jebsen Centre for Psychosis Research, Department of Clinical Science, University of Bergen, Bergen, Norway; 8 Dr. E. Martens Research Group of Biological Psychiatry, Center for Medical Genetics and Molecular Medicine, Haukeland University Hospital, Bergen, Norway; 9 Department of Neuroscience, Norwegian University of Science and Technology (NTNU), Trondheim, Norway; 10 Department of Radiology, St. Olav University Hospital, Trondheim, Norway; 11 Department of Psychology, University of Oslo, Oslo, Norway; 12 NORMENT, KG Jebsen Centre for Psychosis Research, Division of Mental Health and Addiction, Oslo University Hospital, Oslo, Norway; 13 NORMENT, KG Jebsen Centre for Psychosis Research, Division of Mental Health and Addiction, Oslo University Hospital & Institute of Clinical Medicine, University of Oslo, Oslo, Norway; 14 Department of Psychiatry, University of California San Diego, La Jolla, School of Medicine, 9500 Gilman Drive, La Jolla, CA 92037 USA; 15 Center for Human Brain Activity Mapping, University of California San Diego, School of Medicine, 3510 Dunhill Street, San Diego, CA 92121 USA

## Abstract

Despite great interest in using magnetic resonance imaging (MRI) for studying the effects of genes on brain structure in humans, current approaches have focused almost entirely on predefined regions of interest and had limited success. Here, we used multivariate methods to define a single neuroanatomical score of how William’s Syndrome (WS) brains deviate structurally from controls. The score is trained and validated on measures of T1 structural brain imaging in two WS cohorts (training, *n* = 38; validating, *n* = 60). We then associated this score with single nucleotide polymorphisms (SNPs) in the WS hemi-deleted region in five cohorts of neurologically and psychiatrically typical individuals (healthy European descendants, *n* = 1863). Among 110 SNPs within the 7q11.23 WS chromosomal region, we found one associated locus (*p* = 5e–5) located at *GTF2IRD1*, which has been implicated in animal models of WS. Furthermore, the genetic signals of neuroanatomical scores are highly enriched locally in the 7q11.23 compared with summary statistics based on regions of interest, such as hippocampal volumes (*n* = 12,596), and also globally (SNP-heritability = 0.82, se = 0.25, *p* = 5e−4). The role of genetic variability in *GTF2IRD1* during neurodevelopment extends to healthy subjects. Our approach of learning MRI-derived phenotypes from clinical populations with well-established brain abnormalities characterized by known genetic lesions may be a powerful alternative to traditional region of interest-based studies for identifying genetic variants regulating typical brain development.

## Introduction

The morphology of an adult brain represents a holistic snapshot of a unique neurodevelopmental history; its variations are an accumulation of dynamic processes working in concert with few constraints^1^. Different brain regions share the same original sets of proto-structures emerging from interactive molecular signaling programs during early embryonic stage. Post-natal brain growth, myelination, and subsequent regressive processes leading to mature functional circuits provide further overlap in the processes giving rise to adult brain morphology. These developmental processes, furthermore, are guided by distributed patterns of gene expression, interactions with the environment, and operate under spatial constraints imposed by the cranium that may link the morphology of various parts of the adult brain^1,2^. Consequently, the perturbation of a developmentally critical gene often results in diverse morphological abnormalities not limited to a single brain region^3–5^. Given this, it is reasonable to expect that variability interjected into neurodevelopment via a genetic variant may not only contribute to variability in the MRI-derived morphology of a single delineated brain region, but also to covariance among multiple regions^2^.

However, genetic studies of neuroanatomy using magnetic resonance imaging (MRI) continue to prioritize morphological measures on specific landmark-defined brain regions, such as the volumes of subcortical nuclei^6^ or average thickness of cortical parcellations^7^. Although this approach captures some genetic effects of structural variations, it bypasses the fact that the morphological state of an adult brain is the sum of previous developmental processes across brain regions. These landmark-defined regions of interest (ROIs) therefore may have lost genetically relevant information by ignoring co-varied components, while concurrently introducing irrelevant variance by combining measures from genetically unrelated neighbors^8^.

The limitations of this ROI approach are most evident in the context of studying effects on neurodevelopment, as the age-dependent processes have been shown to consist of a gradient spreading across the cortical surface without a discernable relationship to traditional anatomical landmarks^9^. Past efforts to redefine the imaging phenotypes beyond landmark-based ROIs include learning a sparse representation from patients with Alzheimer’s disease^8^ or redrawing ROIs based on the genetic correlations from twin studies^7,10^. These methods can be conceptualized as projecting the multidimensional measures of MRI onto a lower dimensional axis while filtering out components irrelevant to the genetic signals. Such methods have seldom focused, however, on neurodevelopmental disorders, such as Williams Syndrome (WS), that have larger neuroanatomical impacts and more finite candidate genetic regions attributable to the neuroanatomical differences. Since statistical power is the most critical factor for identifying genes through associations^11^, a redefined MRI measure that contains more relevant genetic signals and reduces the burden of multiple comparisons can greatly facilitate the discovery of neurodevelopmental genes.

WS is a multi-systemic disorder caused by hemi-deletion of roughly 27 genes on chromosome 7, resulting in cardiovascular morbidities, intellectual impairment, and hypersociability^12,13^. Besides a decrease of about 11% in brain size, patients with WS have aberrant regionalization of cortical surfaces as assessed with brain MRI, particularly in superior parietal regions and the orbitofrontal cortex^14–18^. Animal models have suggested *GTF2IRD1*, a gene-encoded general transcription factor, as one of the most promising candidate genes for neuroanatomical differences in WS^4,19–21^. Genetic perturbations on *GTF2IRD1* have recently been associated with dog friendliness toward humans^22^. Despite such findings in animal models, associations of this gene with brain or behavioral phenotypes in the healthy human population are lacking^6^. Without association studies on brain phenotypes in healthy human populations, it remains unclear whether common genetic variants on those genes have an impact on typical brain development.

Here, we describe a novel two-pronged approach to capturing genetic effects on neurodevelopment. First, using one single score to represent the global neuroanatomical variations, and a candidate genes approach by examining only the WS region, we limit the effect-size requirements imposed by Bonferroni correction. Second, and more important, we increase the sensitivity of the anatomical phenotype by using a single derived score calculated from multidimensional MRI measures. In our previous work, we derived a single global measure that characterizes how WS brains are structurally different from controls, across multiple parameters in multiple locations^23^. In this study, we demonstrate that the WS neuroanatomical score can be regarded as an MRI endophenotype, enriched in genetic information pertaining to neurodevelopment. By applying the neuroanatomical scores to five imaging genetic cohorts with brain MRI and single nucleotide polymorphisms (SNP) data (*n* = 1863 healthy European descent), we demonstrate, for the first time, that a common variant in *GTF2IRD1* is associated with variation in brain structure (Bonferroni corrected *p* = 0.023). The genetic signals are more enriched than traditionally defined ROI and have significantly high SNP-heritability (*h*^2^ = 0.82, se = 0.25, *p* = 5e−4). Our results provide a proof of concept for the strategy of using multivariate structural measures as a derived intermediate phenotype for genetic association studies.

## Materials and methods

### Healthy imaging genetics cohorts

We selected 1863 healthy imaging genetics subjects from five independent cohorts: 184 from the Alzheimer’s Disease Neuroimaging Initiative (ADNI)^24^, 653 from the Nord-Trøndelag Health Study (HUNT)^25^, 325 from the Norwegian Cognitive NeuroGenetics (NCNG)^26^, 250 from the Thematically Organized Psychosis study (TOP)^27^, and 451 from the Pediatric Imaging Neurocognition and Genetics Study (PING)^28^. From each study, only healthy, unrelated, European-ancestry subjects were retained for analysis. Because the WS neuroanatomical scores were nevertheless trained on an adult WS cohort^23^, the residual confounding of age effect might have an impact on the association. Given that the PING study contains the youngest individuals across all cohorts, we further stratified the PING sample into two subcohorts, one for those ages 16 years and older, and the other for those younger than 16. The cut-point 16 years old is decided based on previous studies that found most of the developmental changes of structural measures asymptote by the age of 16^29,30^. Each study collected 3D T1 MRI images according to comparable acquisition protocols and was processed with the same FreeSurfer reconstruction protocols. The processing protocols include bias correction, registration, segmentation, and 3D surface reconstruction, as implemented in FreeSurfer^29,31^. Studies using the same five imaging genetic cohorts show genetic factors can be consistently estimated, demonstrating the success in protocol homogenization despite differences in scanners and recruiting sites^7^. Whole-genome genotypes were imputed according to the same Mach/Minimac procedure using the 1000 Genomes Project as a reference. Estimated dosages of 110 SNPs falling within the WS hemi-deletion region (chromosome 7q11.23, 72Mb–74Mb, hg19) were imputed with good quality in all cohorts and selected for analysis. Demographics and detailed summaries of data acquisition and processing for each cohort are presented in the Supplementary materials.

### WS neuroanatomical scores

**Fig. 1:**
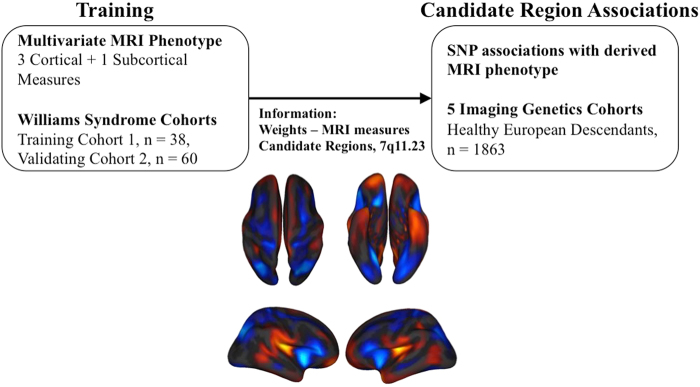
Flow chart of the study design. The first stage of the analysis (Training) was deriving neuroanatomical scores based on case-control data, using a methodology which has been published elsewhere^24^. The second stage of the analysis (Candidate Region Associations) is the focus of this paper, wherein we directly apply the neuroanatomical scores from large-scale imaging genetic cohorts without further calibration of the model parameters

We used a penalized regression model to calculate WS neuroanatomical scores given individuals’ MRI measures. Full details of the training and validation of the model have been published elsewhere^24^. Briefly, 3D T1 MRI images were obtained for 22 WS patients and 16 healthy controls. A multivariate regularized logistic regression was trained to discriminate WS patients from healthy controls on the basis of 30,760 predictors, including estimated cortical surface area^10^, cortical surface geometry^30^, and sulcal depths^16^ for each of 5124 reconstructed vertices and the volumes of 16 subcortical structures^31^. When the model was trained in WS cohort, intra-cranial volume (ICV) was used as a covariate to ensure overall brain size was not driving the classification. Therefore, the WS neuroanatomical score is capturing the subtle morphological reorganization of the WS brain. For each subject in our healthy imaging genetics cohort, we applied the resulting discriminative weights to the same neuroimaging feature space, summarizing this high-dimensional data with a single, composite neuroanatomical score reflecting morphological variations on the axis between healthy individuals and patients with WS. Figure 1 illustrates the flowchart of the analytic strategy and visualization of the weights for contributing neuroimaging measures to the final composite scores. Weights of each imaging measure included in the analyses can be found in Supplementary Figure 1.

### Candidate Region Association analysis

Each imputed SNP dosage was regressed against the WS neuroanatomical score while controlling age, age squared, gender, and the first seven principal components of genetic ancestry as potentially confounding covariates. Although previous analyses from our group had shown consistent estimation of genetic effects across five cohorts^7^, we used meta-analysis to account for potential bias resulting from scanner differences. We estimated the SNP effects in each cohort separately and combined them post-hoc according to an inverse variance weighted meta-analysis implemented in PLINK. To account for multiple comparisons, we used a Bonferroni adjustment for the 110 linked SNPs. Our significance threshold was set to *p* < 0.05/110 = 4.5e−4, conservatively controlling for 110 correlated tests. We then used CAVIAR to determine which SNP is the potential causal variant^32^.

### Local enrichment and global SNP-heritability

To demonstrate the enrichment of local genetic signals with newly defined WS neuroanatomical scores, we used the quantile–quantile plots comparing –log_10_(*p*) between our SNP associations in the WS chromosomal regions and summary statistics obtained from the traditional ROI approach as reported by the ENIGMA consortium (*n* = 12,596)^6^. Despite the scale of our cohorts, the sample size is considered modest in the context of genome-wide association studies. Therefore, to avoid under-powered genome-wide analyses while quantifying the global genetic signals of WS neuroanatomical score, we used Genome-wide Complex Trait Analysis (GCTA)^33^ to estimate the variance explained by all of the SNPs on the entire genome (i.e., the SNP-heritability). The genetic relationship matrix is calculated for all cohorts, using GCTA, and then the SNP-heritability is derived while controlling for age, age squared, gender, cohort membership, and the first seven principal components of genetic ancestry.

## Results

The training and validating of WS neuroanatomical scores have been published elsewhere^24^. In short, the derived neuroanatomical scores robustly distinguished WS from other groups in both the training set (leave-one-out cross-validation area under curve as 100%) and the validating set (area under curve as 100%). The composite WS score significantly mediates the cognitive differences between cases and controls, especially tests quantifying social behaviors^24^. Having derived this multivariate measure which characterizes WS, we then applied the score to healthy imaging genomic cohorts. Each healthy individual’s MRI measures were combined into one single score given the derived weights of WS neuroanatomical score (Supplementary Figure 1). The score of cohort members is normally distributed (mean: 0.6, SD: 0.09) and not correlated with genetic ancestry (absolute Pearson correlations <0.2, *p* > 0.05). None of the cohort members were determined as patients with WS, and none met the anatomical criterion for WS we derived in our earlier work^24^.

**Fig. 2:**
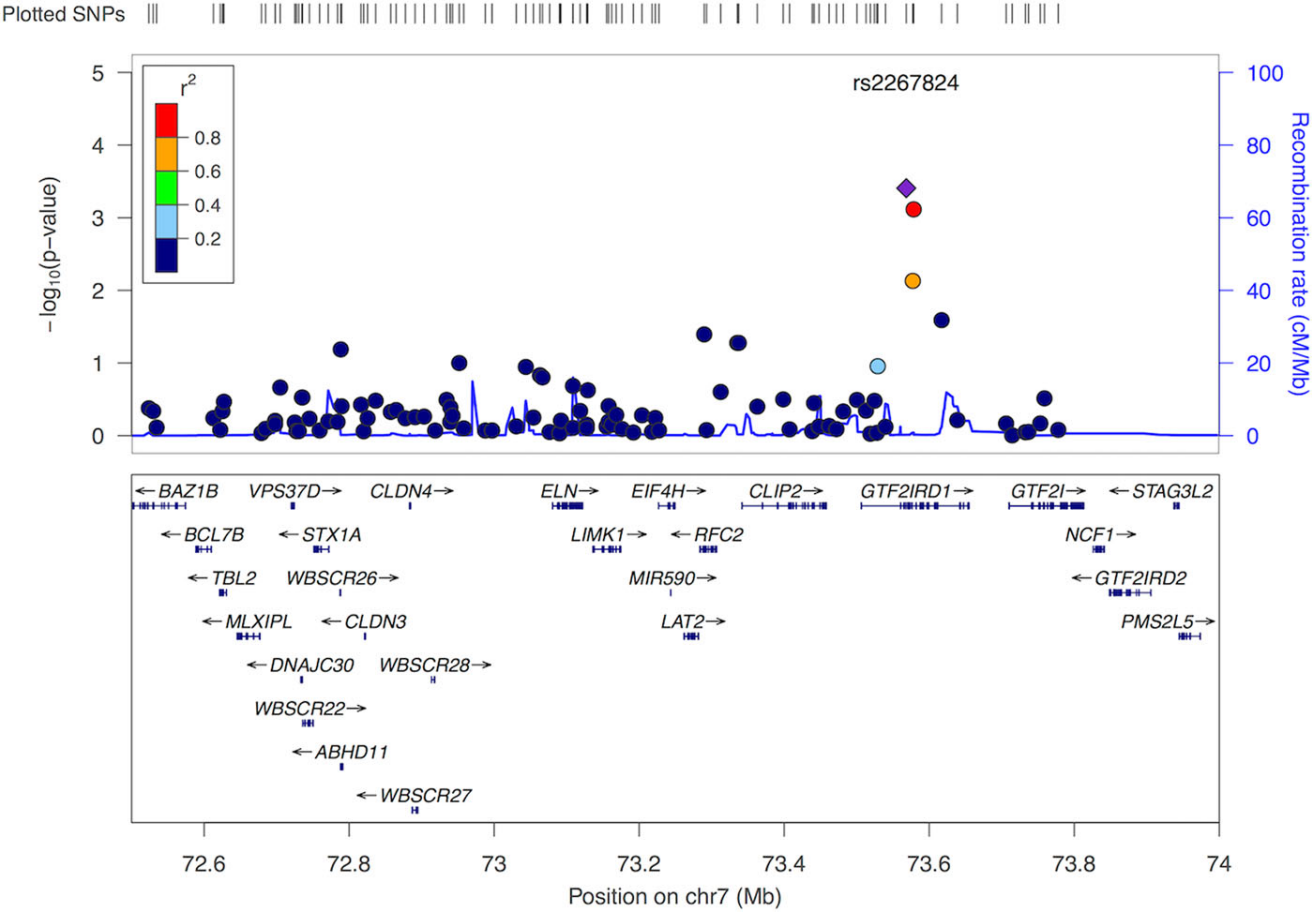
Regional plot of the associations between SNP dosage and WS neuroanatomical scores. The results of 110 SNP associations were plotted against gene annotations and physical positions. The coloring of each SNP represents the linkage disequilibrium with the top SNP, rs2267824

**Fig. 3:**
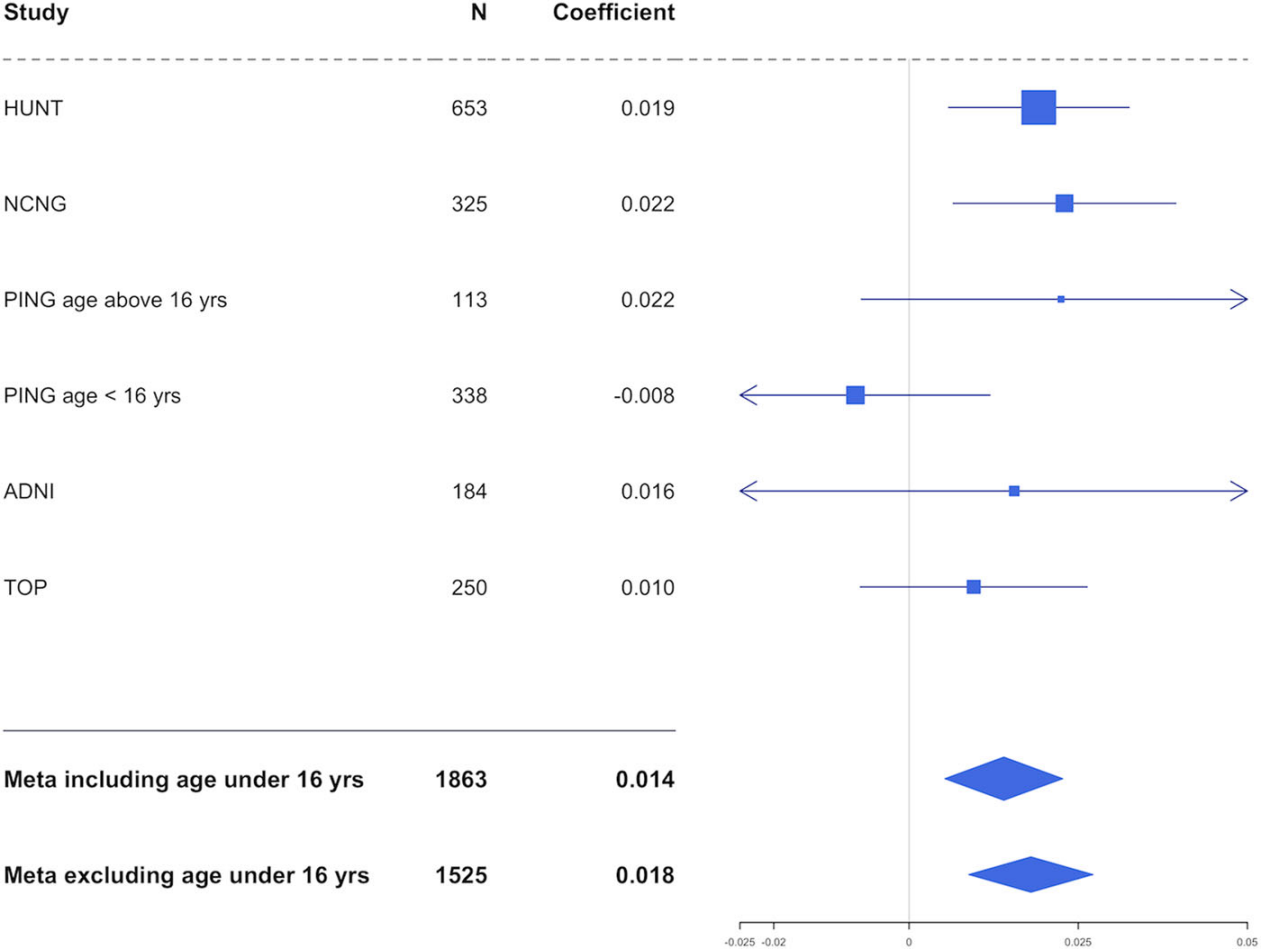
Meta-analysis and stratified analyses of the associations with rs2267824. The reference allele is set as C and the coefficients are presented in arbitrary units, as the WS neuroanatomical scores were similarity measures range from 0 to 1

The associations between SNPs and neuroanatomical score in imaging genomic cohorts are shown in Fig. 2 and Fig. 3. One locus containing three SNPs located at *GTF2IRD1* showed statistical significance after Bonferroni correction (Fig. 2, top SNP, rs2267824, *p* = 2.0e−4). Effect sizes of the associated SNP were consistent across cohorts (Fig. 3) except for the cohort with individuals younger than 16 years old. After excluding individuals younger than 16 years old, the association of rs2267824 became stronger (reference allele: *C*, coefficient: 0.018, *p* = 5e−5). CAVIAR confirmed that the region contains one single locus and rs2267824 was the potential causal variant. In addition, one SNP within 250 kb of *FZF9* showed nominal significance (rs2237280, *p* = 0.00627).

**Fig. 4:**
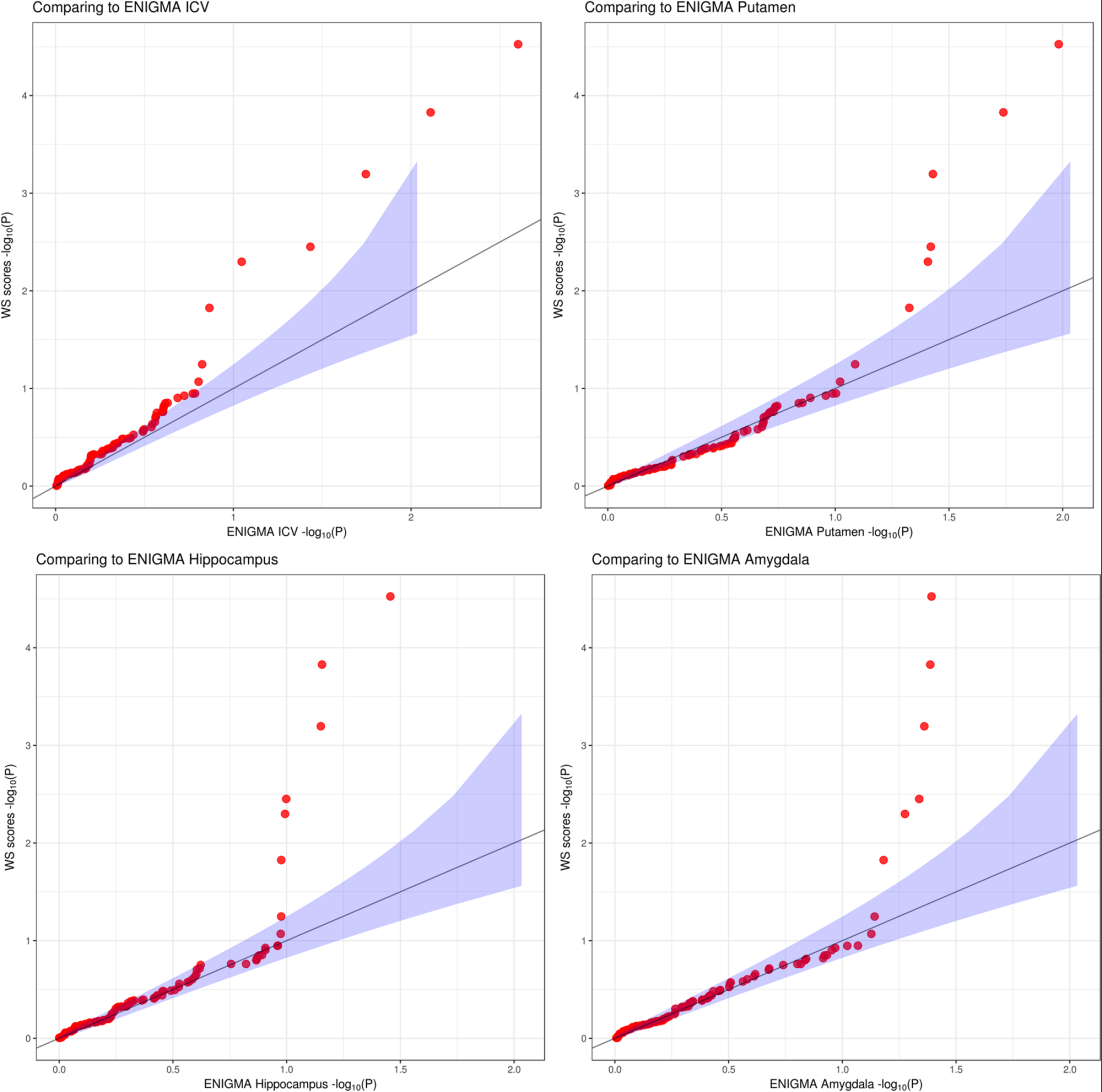
Enrichment of genetic signals using composite neuroanatomical scores. Quantile–quantile plots compare our results and summary statistics from the ENIGMA study^6^. Only SNP associations from the WS chromosomal regions were included in this analysis. To demonstrate the local enrichment, we plot the quantiles of –log_10_(*p*) from SNP associations using neuroanatomical score against the quantiles of –lgo_10_(*p*) from SNP associations using particular anatomical volumes in the ENIGMA. Here, *p* values from a particular anatomical volume in the ENIGMA study across 30,717 individuals are ranked on the X-axis whereas the WS-composite score in our 1863 individuals are on the Y-axis. Different panels compare associations between SNPs of WS chromosomal regions to associations in ENIGMA to different anatomical ROIs: upper left, intra-cranial volumes (ICV); upper right, putamen volumes; lower left, hippocampal volumes; lower right, amygdala volumes. Note that ICV and putamen volume decreases are some of the most common neuroanatomical features in WS^12,17,18^. Although ENIGMA had almost a 10-fold larger sample size than our current study, the genetic signals were enriched in our analyses as the tail of the quantile–quantile plots (red dots) significantly deflected upward from the expected null (solid black line with confidence interval in blue shades)

The quantile–quantile plots compared with associations from the ENIGMA study demonstrated significantly enriched genetic signals in the WS chromosomal regions when using the WS neuroanatomical score (Fig. 4). In terms of global genetic signals, the WS neuroanatomical score has high heritability (*h*^2^ = 0.82, se = 0.25, *p* = 5e−4) despite the fact that less than 1% of phenotypic variation can be explained by the potential causal SNP, rs2267824.

## Discussion

Here we demonstrate that the WS neuroanatomical score can be regarded as an MRI endophenotype, enriched in genetic information pertaining to neurodevelopment. By applying the neuroanatomical score to five imaging genetic cohorts, we show that a common variant in *GTF2IRD1* is associated with variation in brain structure. The genetic signals were more enriched than traditionally defined ROI and have significantly high SNP-heritability. Our results provide a proof of concept for the strategy of using multivariate structural measures as a derived intermediate phenotype for genetic association studies. An optimized multivariate MRI procedure defines the intermediate phenotype that can accurately capture the continuous nature of the underlying brain variations, thus providing greater power for detecting genetic associations.

The associations between *GTF2IRD1* and the WS neuroanatomical score support a critical role of this general transcription factor for normal brain development, and specifically for one of the characteristic personality traits of WS. WS has a unique neuroarchitecture compared to other developmental disorders with intellectual impairment, but few studies have tied anatomical changes to strikingly heightened social behavior^12–18,23^. Previous case studies of partial hemi-deletions in WS indicate that the region telomeric to 7q11.23, which includes *GTF2IRD1*, is crucial for the changes in social behaviors characteristic of WS^4,20,34^. Animal models also support the role of *GTF2IRD1* in brain development^4,19,21^. In particular, a recent study on dog friendliness found the genetic variations on *GTF2IRD1* and *GTF2I* were positively selected for the tendency to socially engage with humans^22^. Together with these results, our findings provide converging evidence for the role of *GTF2IRD1* in human brain development and social cognition.

The associations with *GTF2IRD1* are not consistent across age: the PING sample with age under 16 years old did not show significant associations between WS neuroanatomical score and *GTF2IRD1*. While many factors can lead to this null-association among younger cohort, one possibility is that the developmental genetic effects need to accumulate over time to be detectable. Although the WS neuroanatomical score was validated with a WS child cohort^23^, the score variations are limited among children with typical development, which is the case for the PING sample. Although our current sample size is too small to systematically examine possible age-dependent genetic effects on structural neuroimaging measures, they may become feasible with the large-scale imaging genetic studies now becoming available.

It is likely that other genes also affect the neuroanatomical profiles we defined here, and they may act synergistically in producing the observed phenotype. For example, a study of neuron-like cells derived from stem cells in WS demonstrated reduced neuron proliferation and enhanced dendritic elaboration resulting from perturbation on *FZD9*^35^. As our associations found a suggestive signal located at *FZD9*, although much weaker than the main *GTF2IRD1* effects, it nevertheless jointly contributed to the variations in neuroanatomical profiles. This interpretation is supported by the effects of partial hemi-deletions, which spare the *FZD9* gene^23,35^. We found that although WS neuroanatomical scores increased among these subjects, it is much weaker than in those with a typical hemi-deletion^23^. Further evidence for synergistic effects was found in studies implicating both *GTF2IRD1* and *FZD9* in the Wnt pathway, a well-researched signaling pathway that has been implicated in stem cell control and neuroplasticity^3,34,36^.

In addition, we found significantly high heritability of the observed variations in our defined neuroanatomical score, indicating polygenic contributions. Although the neuroanatomical scores were highly specific to WS status among patient groups^23^, the variations in scores among healthy adults can represent the accumulation of multiple developmental processes with diverse genetic perturbations, each with small effects. This phenomenon is compatible with the theory of the modularized genetic networks in which canalized phenotypes, e.g., typically developed brains, can tolerate many small genetic perturbations unless genetic hubs are drastically disturbed^5,37^. In this framework, the WS deletions would represent a large perturbation of a neurodevelopmental process which in typical developed individuals only shows small variations attributable to regulatory genes across the genome. Although our WS neuroanatomical scores were enriched for WS relevant genetic effects, it nevertheless characterized an underlying canalized developmental process. Using our analytic strategy with diverse genetic developmental disorders may provide further insight into this enduring question about phenotype–genotype mapping.

In sum, our results provide further support for the role of *GTF2IRD1* in the WS phenotype and a proof of concept for deriving multivariate MRI phenotypes for genotype–phenotype studies. This strategy may prove useful in other neurodevelopmental disorders that typically have restricted genetic deletions or alterations. In addition, more accurate measurement of the neuroanatomical phenotype should also provide greater power for genetic studies of diseases such as schizophrenia and autism spectrum disorders where the genetic basis is distributed across the genome, and should ultimately facilitate the discovery of other mediating paths from genes to disorders.

## Electronic supplementary material

Supplemental text

Figure S1
