# Supplementary material for: Williams Syndrome neuroanatomical score associates with GTF2IRD1 in large-scale magnetic resonance imaging cohorts: a proof of concept for multivariate endophenotypes
Source: Transl Psychiatry. 2018 Jun 8;8:114. doi: 10.1038/s41398-018-0166-y (PMC5993783; doi:10.1038/s41398-018-0166-y)
Supplement: Supplementary file 1 — Supplemental text [file 41398_2018_166_MOESM1_ESM.docx]

**Williams Syndrome neuroanatomical score associates with *GTF2IRD1* in large-scale magnetic resonance imaging cohorts: a proof of concept for multivariate endophenotypes**

**Supplementary Information**

**Participants**

Alzheimer’s Disease Neuroimaging Initiative (ADNI)

Of 793 subjects from ADNI database, we included 184 healthy controls in our analyses. The excluded samples are 161 individuals with Alzheimer’s Disease and 216 individuals with Mild Cognitive Impairment. Among 216 participants as controls from ADNI database, we excluded 32 individuals who either have poor imaging qualities or genotyping calls. Demographic characteristics of those included ADNI individuals can be found in Supplemental Table 1. Genotyping was performed with the Illumina Human610-Quad BeadChip. Magnetic resonance imaging (MRI) of brain were collected on 1.5-T scanners at multiple centers across the United States. Raw images were downloaded from the ADNI data page of the public ADNI site at the LONI (http://adni.loni.usc.edu).

Nord-Trøndelag Health Study (HUNT)

Of 842 individuals from HUNT, we included 653 individuals who have good quality of MRI scans, genotyping calls, and unrelated to other individuals involved in the study. All of the enrolled individuals were ethnically European. Age and gender distribution can be found in Supplemental Table 1. Genotyping was based on the Illumina Omni 2.5M BeanChip array. MRI scans were performed with a General Electric Signa HDx 1.5 T scanner.

Norwegian Cognitive NeuroGenetics (NCNG)

NCNG includes 325 individuals with non-missing genotyping and MRI scans. All participants were ethnically European. Age and gender distribution can be found in Supplemental Table 1. Genotyping was based on the Illumina Human610-Quad BeadChip

. MRI scans were performed using several different scanners that detailed protocols can be found at Espeseth, T. et al.^23^.

Thematically Organized Psychosis study (TOP)

Of 1579 subjects from TOP study, we included 250 individuals who were neither diagnosis with schizophrenia nor bipolar disorders. All included individuals were ethnically European. Detailed demographics of those included individuals can be found in the Supplemental Table 1. Genotyping was performed on the Affymetrix 6.0 array. MRI scans of brain were performed with a 1.5 T Siemens Magnetom Sonata scanner equipped with a standard head coil.

Pediatric Imaging Neurocognition and Genetics Study (PING)

Of 1406 samples from PING database, we included 451 individuals who have good quality of genotyping calls, MRI scans, and are ethnically European. The data were obtained from public available database (http://ping.chd.ucsd.edu/) and the detailed demographic information of included individuals can be found in the Supplemental Table 1. Illumina Human660W-Quad BeadChip was used for genotyping; while T1-weighted MRI data were collected on 3-T scanners at nine sites across the United States. Specific MRI scanner protocols are available at the PING study website (http://ping.chd.ucsd.edu/).

**Supplementary Table 1. Demographics of included participants from five imaging genetic cohorts**

|  |  | Age | | | Gender |
| --- | --- | --- | --- | --- | --- |
| Cohorts | n | Mean (yrs) | SD | Range | Percentage (Male) |
| ADNI | 184 | 76.1 | 5.1 | 60 – 90 | 54% |
| HUNT | 653 | 58.4 | 4.2 | 50 – 66 | 44% |
| NCNG | 325 | 51.8 | 16.7 | 19 – 79 | 31% |
| TOP | 250 | 35.5 | 9.7 | 18 - 59 | 54% |
| PING | 451 | 12.2 | 4.9 | 3 – 21 | 53% |

**Supplementary Figure 1. Visualization of the weights for deriving the WS neuroanatomical scores.** Three types of cortical surface measures were included: surface area, sulcal depth, and surface geometry. Subcortical regions not illustrated here included putamen, hippocampus, and amygdala volumes. Due to the penalty we imposed for the large number of imaging measures, the weights were all small, with maximum values less than 5x10^-4^.
