# Supplementary figures and images for: Williams Syndrome neuroanatomical score associates with GTF2IRD1 in large-scale magnetic resonance imaging cohorts: a proof of concept for multivariate endophenotypes
Source: Transl Psychiatry. 2018 Jun 8;8:114. doi: 10.1038/s41398-018-0166-y (PMC5993783; doi:10.1038/s41398-018-0166-y)

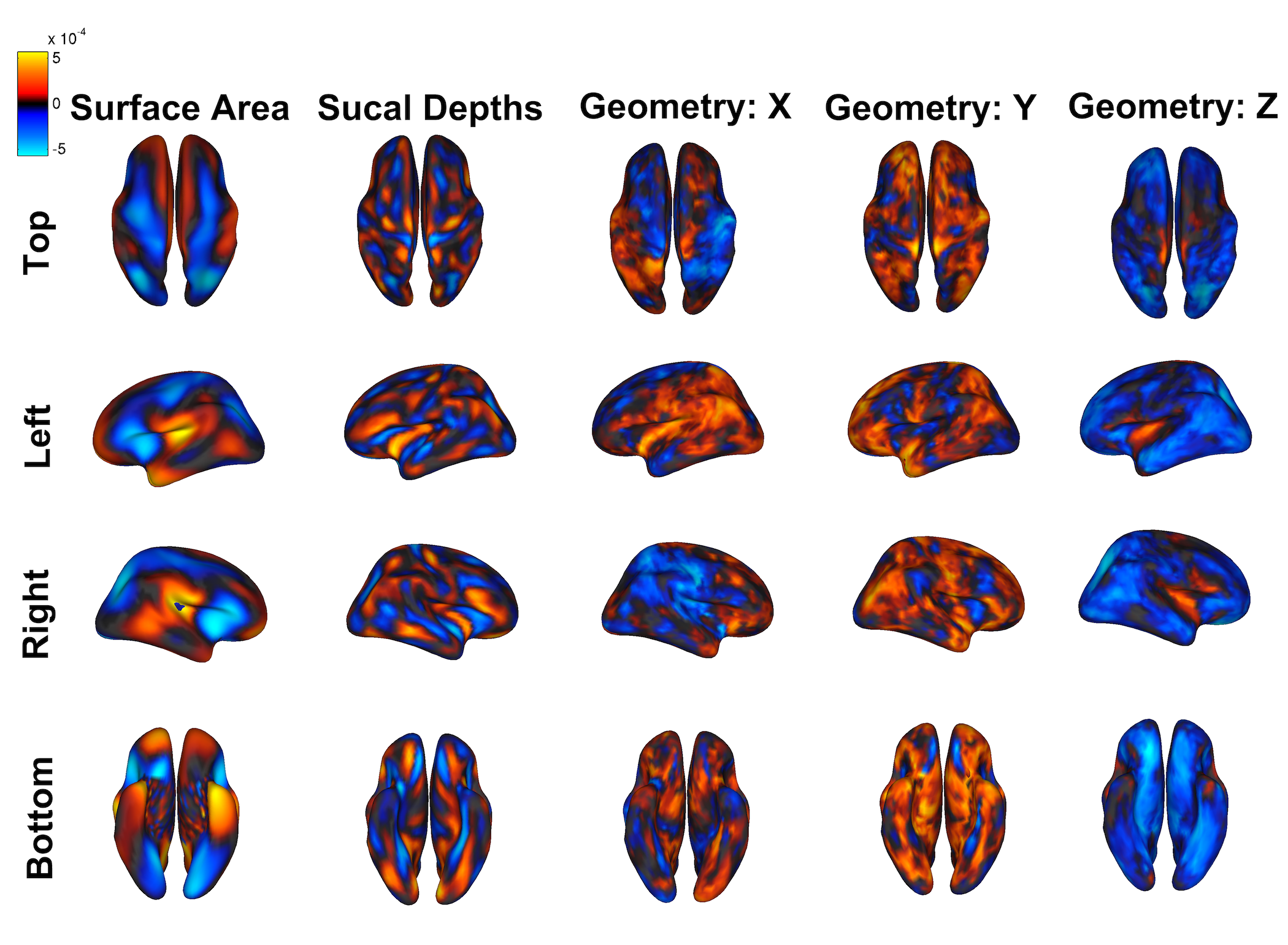

Supplement: Supplementary file 2 — Figure S1 [file 41398_2018_166_MOESM2_ESM.tif]
